# Multi-Composite Bioactive Osteogenic Sponges Featuring Mesenchymal Stem Cells, Platelet-Rich Plasma, Nanoporous Silicon Enclosures, and Peptide Amphiphiles for Rapid Bone Regeneration

**DOI:** 10.3390/jfb2020039

**Published:** 2011-06-21

**Authors:** Matthew B. Murphy, Daniel Blashki, Rachel M. Buchanan, Dongmei Fan, Enrica De Rosa, Ramille N. Shah, Samuel I. Stupp, Bradley K. Weiner, Paul J. Simmons, Mauro Ferrari, Ennio Tasciotti

**Affiliations:** 1 Department of Nanomedicine and Biomedical Engineering, The Methodist Hospital Research Institute, Houston, TX 77053, USA; E-Mails: mbmurphy@tmhs.org (M.B.M.); rmbuchanan@tmhs.org (R.M.B.); dfan@tmhs.org (D.F.); ederosa@tmhs.org (E.R.); mferrari@tmhs.org (M.F.); 2 Centre for Stem Cell Research, The University of Texas Health Science Center at Houston, Houston, TX 77053, USA; E-Mails: blash007@hotmail.com (D.B.); paul.j.simmons@uth.tmc.edu (P.J.S.); 3 Department of Biomedical Engineering, The University of Texas at Austin, Austin, TX 77053, USA; 4 Department of Materials Science and Engineering, Northwestern University, Chicago, IL 60086, USA; E-Mails: ramille-shah@northwestern.edu (R.N.S.); s-stupp@northwestern.edu (S.I.S.); 5 Department of Orthopaedics, The Methodist Hospital, Houston, TX 77053, USA; E-Mail: bkweiner@tmhs.org

**Keywords:** bone regeneration, tissue engineering, composite scaffold, mesenchymal stem cells, platelet-rich plasma, nanoporous silicon, peptide amphiphiles

## Abstract

A novel bioactive sponge was created with a composite of type I collagen sponges or porous poly(ε-caprolactone) (PCL) scaffolds, platelet-rich plasma (PRP), BMP2-loaded nanoporous silicon enclosure (NSE) microparticles, mineralizing peptide amphiphiles (PA), and mesenchymal stem cells (MSC). Primary MSC from cortical bone (CB) tissue proved to form more and larger colony units, as well as produce more mineral matrix under osteogenic differentiation, than MSC from bone marrow (BM). Coating pre-treatments were optimized for maximum cell adhesion and mineralization, while a PRP-based gel carrier was created to efficiently deliver and retain MSC and microparticles within a porous scaffold while simultaneously promoting cell recruitment, proliferation, and angiogenesis. Components and composite sponges were evaluated for osteogenic differentiation *in vitro*. Osteogenic sponges were loaded with MSC, PRP, PA, and NSE and implanted subcutaneously in rats to evaluate the formation of bone tissue and angiogenesis *in vivo*. It was found that the combination of a collagen sponge with CB MSC, PRP, PA, and the BMP2-releasing NSE formed the most bone and was most vascularized by four weeks compared to analogous composites featuring BM MSC or PCL or lacking PRP, PA, and NSE. This study indicates that CB MSC should be considered as an alternative to marrow as a source of stem cells, while the PRP-PA cell and microparticle delivery system may be utilized for diverse tissue engineering applications.

## 1. Introduction

With more than one million non-union fractures treated each year in the United States and many presenting significant challenges to repair, a significant demand persists for functional and affordable synthetic systems for *in vivo* bone regeneration and fracture repair [1,2,3]. Tissue engineering strategies for bone repair endeavor to create alternative but functional constructs to guide new bone formation [4]. Extensive research has been conducted on the *in vitro* interactions of biomaterials and bone progenitor cells in order to characterize their potential for *in vivo* bone regeneration. [5,6,7,8,9,10,11]. Ideally, a tissue engineering construct will support the formation of new bone at a similar rate to its own biodegradation, eliminated the necessity of secondary surgeries [12]. Scaffolds are also required to be porous, allowing for vascular integration for the transport of nutrients and waste to cells within the defect. A novel and sometimes under-utilized strategy in the biomaterials field is the combination of previously successful materials to form a novel multi-functional composite to trigger the rapid formation of bone through multiple simultaneous mechanisms. We previously described benefits of varied stem cell populations, bioactive factors, and biomaterials towards the repair of critically sized bone defects [13]. In this study, we have designed a multi-composite bioactive sponge based upon a highly porous scaffold loaded with two classes of mesenchymal stem cells, mineralizing peptide amphiphiles (PA), platelet-rich plasma (PRP), and growth factor delivering nanoporous silicon enclosure (NSE) microparticles for accelerated bone regeneration.

We selected two types of scaffolding materials based on their intrinsic properties and previous success in bone tissue engineering. Type I collagen, the major organic component of bone matrix, comprises almost 30% of all tissue proteins and serves as a truly natural substrate for tissue in growth [14]. Previous reports state that a recreation of the niche, or native environment, is imperative for correct and optimal function of stem cells within a regenerating or remodeling tissue [15,16]. Mineralized collagen, like that found in bone, has been shown to successfully heal critical size skeletal defects *in vivo* [17]. Poly(-caprolactone) (PCL) is a biocompatible, biodegradable synthetic polymer frequently used a scaffold material in the tissue engineering field [18,19,20]. Like many common biomaterial polymers, PCL is a hydrolytically degradable polyester with slower degradation rates and milder byproducts than poly(lactic-co-glycolic acid) (PLGA) [21,22]. Due to its gentler degradation environment, PCL has shown better cell adhesion and proliferation *in vitro* and better angiogenesis while forming new bone *in vivo* than PLGA or its predecessors (PGA and PLLA), but often lacks the mechanical properties necessary for load-bearing applications [23,24,25,26]. However, the objective of this study was to produce an osteogenic sponge to meet all the biological requirements for rapid bone formation without consideration of compressive or torsional loads experienced in long bones, making collagen and PCL sponges suitable candidate materials. Aside from the stiffness and strength necessary to maintain the scaffold's architecture, mechanical properties of the material was not considered an important factor. These osteogenic sponges are intended to be implanted in conjunction with a rigid fixation device to stabilize fractures while new bone and tissue is created. Non-union fractures can take months to years to heal, so the degradation of the implants was desired to be minimal through the first month *in vivo*.

Bone regeneration requires osteogenic, or bone forming, cells to lay down a protein and mineralized matrix upon a scaffold or template when provided with the proper biological cues. Mesenchymal stem cells (MSC), the most common type of cell for this application, are typically harvested from the bone marrow [27,28,29,30,31,32,33,34,35,36,37]. Autologous (from the same patient) MSC are considered safer, as there is less risk of foreign body recognition and immune rejection, but substantial work has pointed to the immuno-suppressive properties of true MSC such that allogenic (from a different donor) cells may be a viable option in tissue regeneration. The use of allogenic MSC is particularly attractive when dealing with older or sick patients suffering from a scarcity of their own adult stem cells. MSC are defined by their capacity to give rise to bone, cartilage and adipose tissue [38,39,40,41]. Osteogenic differentiation of MSC is regulated by a broad repertoire of cell extrinsic factors including the WNTs, various transforming growth factor-beta super-family members, Notch, Hedgehog and fibroblast growth factors (FGFs) [42]. In common with skeletal development, fracture repair requires the coordination of multiple events such as migration, differentiation, and activation of multiple cell types and tissues [43].

The development of microvasculature and microcirculation are critical for regeneration of bone during fracture repair, without which, the tissue degenerates and dies [44,45]. The bioactive sponge must demonstrate the capacity to: 1. Promote angiogenesis at the site of the defect; 2. Efficiently recruit MSC; 3. Retain the MSC on site; 4. Stimulate their subsequent proliferation and differentiation into load-bearing bone; and 5. Enhance the net formation of bone by minimizing osteoclast-mediated resorption. In support of goals 1–4, we have developed a system of PRP as a delivery agent of cells and growth factor-loaded microparticles that can simultaneously release its natural cocktail of growth factors and chemokines. PRP has been shown to stimulate the production of alkaline phosphatase (ALP, a marker for differentiation) and increase collagen and mineral production [46]. This phenomenon was increased when the MSC were grown on 3D tissue engineering scaffolds in the presence of PRP and osteogenic factors for three weeks [47]. PRP also stimulates the division and growth of stem cells *in vitro* without the use of serum [48,49]. PDGF, FGF, TGF- and other growth factors discharged from platelets promote cell proliferation, while chemokines including SDF-1, RANTES, MIG, and SRPSOX boost bone marrow MSC migration in the direction of the chemical gradient [50,51,52,53,53]. PRP has already proven useful in tissue engineering and orthopedic applications in the treatment of fractures, soft tissue wounds, and sports injuries [47,54,55,56]. Others report that PRP osteogenic factors such as bone morphogenetic protein-2 (BMP2) to successfully induce bone formation *in vivo* [57,58,59].

Self-assembling PA consisting of an outer hydrophilic segment, a hydrophobic alkyl tail, and a beta-sheet forming peptide segment [60]. PA self-assemble through electrostatic molecular interactions due to changes in pH or addition of multivalent ions into 3D structures with nanofeatures 5 to 8 nm in diameter and can be several micrometers in length [61,62]. The resulting nanofibers may display over 1000 bioactive signals per cm^2^ and are useful in innumerous biological applications [63,64,65,66,67,68,69]. Combination of multiple PA monomers bearing different biological activities, such as cell-adhesion sequences or growth factor binding sequences, may be employed to increase the overall functionality of the material [62,66,70,71,72]. Their design can be tailored to promote cell attachment and mineralization as they mimic the native structure of ECM proteins such as collagen [60,68,73,74,75]. PA containing phosphoserine (S(P)) are able to nucleate hydroxyapatite crystals parallel to the nanofibers and calcium phosphate mineralizes throughout the nanofiber network [63]. As they are primarily composed of natural amino acids, fatty acids, and peptide segments, PA present no practical toxicity and a minimum immune or inflammatory response [67,69,76]. Initial *in vivo* studies showed sign of osteogenic repair in bone defects by use of S(P) PA, evidence that this material may be supplement the osteogenic potential of MSC and BMP2 [63,74,77].

Nanoporous Silicon (pSi) has proven clinically successful in multiple therapeutic applications and was previously explored by our group as a multistage delivery system [78,79,80]. The NSE proved to promote hydroxyapatite mineralization *in vitro* and bone formation in animal models [81,82,83,84]. It is osteoinductive, or has the capacity to stimulate primitive stem cells or immature bone cells to grow and mature, forming healthy bone tissue and can serve as a scaffold for MSC growth to conduct bone healing and regeneration [84,85]. Generally, bioactive glass exhibits greater bioactivity than hydroxyapatite ceramics due to the apparent direct bone bonding effect and proposed genetic modulation by the silicon-based beads [86,87]. The bioactivity, biodegradation rates, and biomechanical properties of the particles can be controlled by their method of fabrication. We have demonstrated the tailoring of NSE to fulfill the requirements of complex applications, including drug delivery [80,88,89,90].

While these materials have individually proven beneficial to bone formation, their synergistic effects had not been explored. It was our hypothesis that a combination of these osteoinductive agents (scaffolds, MSC, PRP, PA, and NSE) would prove successful for *in vitro* mineralization and *in vivo* bone formation, and offer a powerful alternative to other bioactive bone sponges clinically available today. Towards this end, stem cells were isolated from bone marrow and cortical bone compartments and tested with various combinations of these materials for maximum cell adhesion, proliferation, differentiation, and bone formation. Additionally, we developed a novel PRP-PA injectable carrier system to load scaffolds with cells and microparticles with great retention.

## 2. Experimental Section

### 2.1. Materials

Collagen I (bovine, type I), poly(-caprolactone) (PCL, Mn 60,000), poly(lactic-co-glycolic acid) (PLGA, Mn 5,500), gelatin (porcine), fibrinogen (bovine), 1-ethyl-3-(3-dimethylaminopropyl) carbodiimide (EDC), N-hydroxy-sulfosuccinimide (NHS), sodium chloride (NaCl), dexamethasone, ascorbate-2-phosphate, sodium phosphate, silver nitrate (AgNO_3_), sodium thiosulfate, alizarin red, toluidine blue, methylene chloride, ethanol, acetic acid, sodium hydroxide (NaOH), formalin, hematoxylin, eosin, and Quant-it PicoGreen DNA assay were purchased from Sigma Aldrich (Sigma Chemical Company, St. Louis, MO, U.S.). Bovine thrombin was purchased from BioPharm Laboratories (Bluffdale, UT, U.S.). A DICA-500 calcium quantitative assay kit was purchased from QuantiChrom (Bioassay Systems, Hayward, CA, U.S.). Phosphate buffered saline (PBS), alpha modified essential medium (MEM), penicillin/streptomycin, glutamax, and sodium pyruvate were acquired from Invitrogen (Carlsbad, CA, U.S.). Fetal bovine serum (FBS, embryonic stem cell grade) was purchased from HyClone (Thermo Fisher Scientific, Logan, UT, U.S.). StemSpan serum-free media was acquired from Stem Cell Technologies (Vancouver, Canada). Recombinant human bone morphogenetic protein-2 (BMP2) and recombinant human fibronectin were purchased from R&D Systems (Minneapolis, MN, U.S.). Platelet-rich plasma was derived from adult human and rat blood in similar manners. Human buffy coats (purchased from Gulf Coast Regional Blood Bank, Houston, TX, U.S.) and Lewis rat blood (acquired via ventricular aspiration after sacrifice of male rodents and collected in heparinized tubes) was centrifuged at 300g without brake for 15 minutes to separate plasma and platelets from red blood cells. The platelet-containing fraction was then spun at 1600 g for 10 minutes in order to pellet platelets out of the plasma. Platelets and RBC counts were performed at each stage with a Sysmex hematology analyzer (model KX-21N, Mundelein, IL, U.S.). The appropriate amount of plasma was re-added to the platelets to achieve a final PRP concentration of 10^6^ platelets/μL. Peptide amphiphiles (PA) were synthesized by solid phase peptide synthesis chemistry. The E_3_ PA and S(P) PA amino acid sequences were C_16_V_3_A_3_E_3_ and C1_6_A_3_L_3_E_2_S(P)G, respectively. Statistical analyses were performed by one-way and two-way analysis of variance (ANOVA) using Origin 8.5 software.

### 2.2. Mesenchymal Stem Cells for Bone Regeneration

Rat bone marrow (BM) and cortical bone (CB) cells were isolated from male Sprague-Dawley *(in vitro* experiments) and Lewis *(in vivo* experiments) rats by the flushing and crushing/enzymatic-digestion (3 mg/mL collagenase + 4 mg/mL dispase in PBS for 1 h at 37 °C) of hind limb bones, respectively. Mononuclear cell fractions were obtained by centrifugation of cell products in Ficoll at a 1:1 ratio of media to Ficoll. The primary cells were then counted and seeded at initial densities of 1000 to 5 × 10^6^ cells/cm^2^ to assess colony forming unit-fibroblast (CFU-F) frequency of the two bulk and purified populations. After 14 days in hypoxic culture (37 °C, 5% O_2_, 5% CO_2_), cells were washed with PBS, fixed and stained with a toluidine blue solution to evaluate the number and size of colonies.

The osteogenic differentiation capacity of these cell populations was tested by seeding early passage (P1 and P2) cells in 24 well plates at a density of 5000 cells per well. Cells were provided standard (MEM, 20% FBS, 1% glutamax, 1% sodium pyruvate, 1% antibiotics) or osteogenic media (standard media supplemented with 10^−8^ M dexamethasone, 10^−4^ M ascorbate-2-phosphate, and 4 mM sodium phosphate) and cultured for 14 days at 37 °C in 5% O_2_ with media changes every 4 days. Groups were then qualitatively assessed by washing wells twice in PBS and sequentially staining for ALP activity and mineralization. Alkaline phosphatase stains were achieved by mixing substrates for ALP (Alkaline phosphatase Substrate Kit III, Vector Laboratories, Burlington CA) in a Tris-HCL buffer which was incubated on the wells in the dark at room temperature for 30minutes. After ALP staining, wells were washed with the Tris buffer, then with PBS before staining for mineralization (Von Kossa). Von Kossa staining was performed by incubating wells in a 5% AgNO_3_ in distilled water solution for 60 minutes under UV light exposure. AgNO_3_ was then removed and replaced with 5% sodium thiosulfate which exchanges cations on the surface. After 30 minutes, the final step involved a counter stain of 2% Alizarin red for one hour with gentle rocking.

### 2.3. BMP2-releasing Nanoporous Silicon Enclosures

BMP2 releasing, PLGA-coated NSE were prepared by loading of BMP2 growth factor in 200 μL PBS into 8 × 10^7^ oxidized pSi in a 2 mL Eppendorf tube. The suspension was mixed vortexing and sonication, and then incubated at room temperature for 2 h to allow the adsorption of BMP2 into pSi pores. The particles were centrifuged (4500 rpm for 5 minutes). The BMP2 loaded pSi were lyophilized and stored at −80 °C. The amount of BMP2 unabsorbed or unloaded was quantified by measuring BMP2 concentration in the supernatant by ELISA assay.

**Figure 1:**
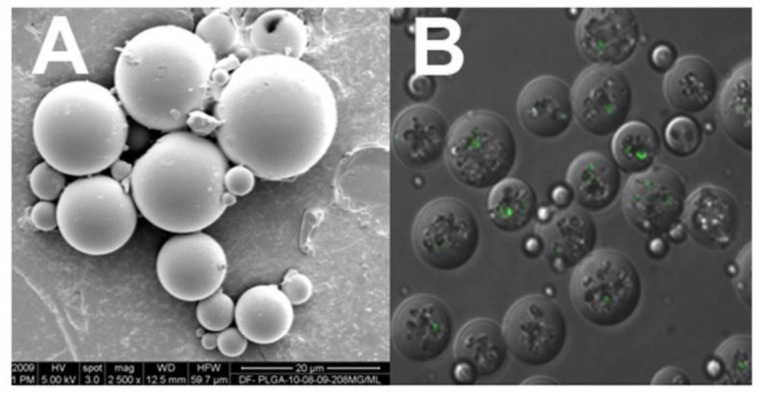
Scanning electron microscopy image of PLGA coated NSE microparticles (**A**), while NSE loaded with FITC-BSA are visible in the core of the particles through fluorescence microscopy (**B**).

The drug-loaded NSE was coated with PLGA using a modified solid-in-oil-in-water emulsion method as mentioned in our previous studies [59]. Briefly, PLGA was dissolved in methylene chloride at concentrations of 20% (w/v). The 8 × 10^7^ BMP2 loaded particles were suspended in 1 mL of PLGA solution and sonicated for 2 minutes. The organic phase containing the pSi was mixed with 3 mL of PVA (2.5% w/v in water) by vortex mixing and sonication. The mixture was cast into 50 mL of mild PVA solution (0.5% w/v in water). The resulting suspension was stirred by a magnetic stirrer at high speed for 2 hours to allow for solvent evaporation. The PLGA/pSi microparticles were washed with three times in Millipore water, lyophilized, and stored at −80 °C. For visual confirmation of the NSE embedded within the PLGA spheres, sample NSE were loaded with FITC-labeled bovine serum albumin (BSA) and imaged by fluorescent confocal microscopy. A complete release study was conducted to measure the drug release kinetics over 30 days (not included in this publication).

### 2.4. Scaffold Fabrication

**Figure 2:**
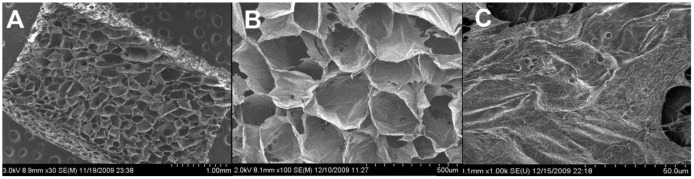
Scanning electron microscopy image of collagen sponge illustrates the interconnected pore network throughout the scaffold (**A**), with uniform pores in the range of 200–500 m, acceptable for vascular and tissue integration for *in vivo* tissue engineering (**B**). Collagen sponges covered with a fine mineralized surface coating after application of S(P)/E_3_ PA and two days of *in vitro* culture in osteogenic media (**C**).

Porous collagen sponges were prepared by the controlled freezing, lyophilization, and crosslinking of a collagen type I slurry. The collagen, in the form of dried fibers, was mixed in 0.05 M acetic acid to a final concentration of 0.5% by weight. The mixture was shear-homogenized in an ice-bath twice for 60 minutes each. The slurry was then de-gassed in vacuum at room temperature for 30 minutes and stored at 4 °C. The solution was cast in and aluminum weighing dish (McMaster Carr, 2 7/8″ diameter) and frozen to −10 °C in tray dryer at an average cooling rate of 0.3 °C/min. Collagen sheets were then lyophilized for at least 36 hours. dehydrothermal (DHT) crosslinked overnight (105 °C in vacuum oven). Using a biopsy punch, individual scaffolds were cut from the dried collagen sheets. Scaffolds were chemically crosslinked in 70% ethanol containing EDC and NHS at a ratio of 1:1:5 EDC:NHS: –COOH (0.0012 mol –COOH/g collagen) for a minimum of two hours. Scaffolds were washed in sterile water and stored at room temperature until use. Scanning electron microscopy (SEM) was used to confirm uniform pores with pore diameters in the range of 200–500 μm (Figure 2A) under this fabrication protocol.

Porous PCL scaffolds were prepared by dissolving the polymer in methylene chloride at a concentration of approximately 150 mg/mL at room temperature. NaCl particles were sieved to obtain crystals in the size range of 200–500 m and dried in a vacuum oven at 105 °C. The dried salt was added to the polymer solution such that the final mixture contained 80% NaCl by weight. The mixture was cast in 1 cm diameter Teflon molds, fixed by solvent exchange in ethanol, and the salt was leached out in an excess of Millipore water over 5 days. Scaffolds were sterilized by ethylene oxide and stored at room temperature until use.

### 2.5. Pre-Mineralization of Scaffolds in Vitro

For enhanced osteointegration and bioactivity of implants, scaffolds were pre-treated by mineralization *in vitro* in osteogenic media with and without E_3_ and S(P) PA coatings. The PA were dissolved in PBS (1% each w/v) and dispensed onto the collagen sponges. The composite scaffolds were immersed in a mineralizing osteogenic medium supplemented with 20 mM CaCl_2_ for 2 days. Mineral was visualized by SEM imaging and calcium-phosphate ratios measured by energy-dispersive X-ray spectroscopy (EDX).

### 2.6. Scaffold Coatings and Injectable Carrier Gel for Cell and Microparticle Retention

To test the cell adhesion properties of candidate coating materials, non-treated, non-cell adherent, bacterial-grade plastic dishes were coated in PBS (negative control), 1% gelatin (by weight) in PBS, 10 g/mL fibronectin, 10% PPP, or 10% PRP. Dishes were refrigerated overnight at 4 °C then washed with PBS. 5,000 CB MSC were seeded in serum-free Stem Span media on each location and allowed one hour at 37 °C for attachment. After incubation, the dishes were washed twice in PBS and 500 L Millipore water was dropped on each seeding location to lyse cells and liberate double-stranded DNA. Aspirates of each DNA solution were analyzed via PicoGreen assay to determine the number of cells attached. Each experimental condition was performed in triplicate (n = 3). Towards translation of *in vitro* studies to *in vivo* application, a comparative study was performed to test differences between species (human and rat) in the adhesive properties of PRP. An adhesion assay was performed as previously described with 0.1, 1, and 10% rat or human PRP. Coated dished were refrigerated overnight and washed with PBS as in the previous study. 2,500 rat CB MSC were seeded in at least three coated locations (n = 3) and incubated for 1 hour at 37 °C. Each dish was washed twice in PBS and cells lysed by addition of 500 L Millipore water and undergoing a freeze-thaw cycle to −80 °C. Cell counts were determined via PicoGreen assay.

To test cell adhesion to three dimensional tissue engineering scaffolds, porous PCL and collagen scaffolds were pre-coated in PBS (negative control), 1% gelatin, 10% PPP, or 10% PRP. 25,000 CB MSC were seeded in a 100 L suspension in StemSpan media onto each scaffold in 24 well cell culture plates. The cell suspension was slowly dropped onto the scaffold, and then placed into an incubator at 37 °C for 5 minutes. Excess solution at the bottom of each well was pipetted and re-applied over the scaffold, with an additional 5 minute incubation at 37 °C. A third and final re-application of the excess cell suspension was distributed onto the scaffolds, followed by 15 minutes of attachment time in the incubator. Each well was supplemented with 1 mL of complete media (20% FBS in αMEM). After one hour, scaffolds were removed from the media, washed twice in PBS, chopped into pieces to increase accessible surface area, and then placed in 1 mL Millipore water and freeze-thawed to lyse cells for DNA isolation. Cell counts were determined by PicoGreen assay and experiments were performed in quadruplicate (n = 4).

Injectable cell carrier gels were utilized to load cells into the pore network of scaffolds. PA gels were fabricated as previously reported. Briefly, a 2% PA solution (50:50 mixture of S(P) and E_3_ peptides) were dissolved in sterile PBS and pH balanced to neutral with 0.1 M NaOH. A second solution was prepared of 100 mM CaCl_2_ in PBS and filtered with a 0.22 m syringe filter. Cells were suspended in the PA solution (50,000 CB MSC/100 L). 100 mL of the each solution was added to each scaffold and allowed to form a self-assembling gel. Aspirating and reapplication of ungelled liquid was performed until no liquid remained. A more extensive type of gel consisted of another two solution system (Table 1). The first solution contained PRP (20% v/v), PA (2% w/v, if included), fibrinogen (3 mg/mL), and cells (50,000/100 L) in PBS. The second solution was comprised of 100 units/mL thrombin and 100 mM CaCl_2_ (if PA are used) in PBS. Growth factor-releasing microparticles may be included in either working solution, but were mixed into Solution A for this study. These solutions were kept separate and warmed to 37 °C, then applied in equal volumes to scaffolds (100 mL of each solution per scaffold). A viscous gel formed immediately and became solid in approximately two minutes.

**Table 1:** Injectable carrier gel components in a two part system.

**Material**	**Solution A**	**Solution B**	**Final Carrier**
PRP	20%	-	10%
Osteogenic PA	2%	-	1%
Fibrinogen	3 mg/mL	-	1.5 mg/mL
CaCl_2_	-	100 mM	50 mM
Thrombin (Human)	-	100 units/mL	50 units/mL
MSC	If applicable		
GF-loaded Microparticles	If applicable		

As a secondary control, 50,000 MSC were seeded into standard tissue culture wells and incubated simultaneously. After 24 h of incubation, the media was removed and scaffolds/cells were washed twice in PBS. Scaffolds containing the carrier gel were treated with 100 μg/mL Proteinase K to break up the fibrin clots and fully liberate cells/DNA after freeze-thaw. Scaffolds were then crushed, exposed to 1 mL Millipore water, and freeze-thawed to ensure cell lysis. Cell counts were determined by PicoGreen assay. All experimental groups were performed in quadruplicate (n = 4). A replicate of each experimental group was prepared using CSFE-labeled cells and Texas Red-labeled NSE to visualize distribution via fluorescent confocal microscopy.

### 2.7. In Vitro Mineralization-Injectable Gel Carrier

To study the ability of MSC to differentiate and mineralize in the presence of PRP, 25,000 CB MSC were gelled in 500 L of the PRP-based gel carrier in 1 cm Teflon molds. Acellular gels were prepared as a control. After 5 minutes of gelation at 37 °C, the composite were transferred to 6 well tissue culture plates and cultured for 21 days in hypoxic conditions (5% O_2_) in standard or osteogenic media. Media was completely changed every 4 days and microscope photographs every 7 days. After 21 days, composite gels were rinsed twice in PBS and then soaked in PBS for 1 hour to remove excess calcium from the media. Gels were then minced with a scalpel. Half of each gel was solublized in 0.1 N acetic acid (calcium quantification) and the other half freeze-thawed in 1 mL Millipore water to −80 °C (cell number counts). Soluble calcium was measured by QuantiChrom DICA-500 assay kit by addition of calcium-binding dyes and UV absorbance at 610 nm. Cell number was quantified by PicoGreen assay.

### 2.8. In Vitro Mineralization—Assembled Scaffolds

*In vitro* osteogenic differentiation assays were performed on porous PCL and collagen scaffolds. Scaffolds were coated in 10% human PRP for 12 h at 4 °C, washed in PBS, then loaded with 200,000 CB or BM MSC with the gel carrier solution (150 L, 10% PRP in media). A description of the experimental groups are listed in Table 2. Groups without PRP were gelled in a PRP-free carrier (final concentration of 1.5 mg/mL fibrinogen and 50 units/mL thrombin in PBS). The “PA only” group (no cells, PRP, or NSE) was loaded by gelling S(P) and E_3_ PA (1% each w/v) with 50 mM CaCl_2_. All composites were allowed to gel for 30 minutes, washed with PBS, then supplemented with osteogenic media for 15 days. Constructs were cultured at 37 °C with 5% O_2_ and media changes every 4 days. All experiments were performed in quadruplicate (n = 4). After 15 days in culture, scaffolds were washed in PBS and lysed in Millipore water for PicoGreen DNA quantification. After cell lysis, composites were decalcified in 0.1 N acetic acid for calcium (mineralization) quantification. Total calcium in the mineral matrix of the differentiating MSC was quantified by QuantiChrom assay via UV absorbance at 610 nm.

**Table 2:** *In vitro* mineralization of PCL and collagen scaffolds receiving combinations of MSC, PA, PRP, and NSE.

**Group Name**	**Cell Type**	**PRP**	**PA**	**NSE**
1. Scaffold	**-**	**-**	**-**	**-**
2. PA only	**-**	**-**	**X**	**-**
3. CB MSC	**CB**	**X**	**X**	**X**
4. BM MSC	**BM**	**X**	**X**	**X**
5. No PRP	**CB**	**-**	**X**	**X**
6. No PA	**CB**	**X**	**-**	**X**
7. No NSE	**CB**	**X**	**X**	**-**

### 2.9. In Vivo Bone Formation and Angiogenesis

#### 2.9.1. Implant Preparation and Loading Efficiency

The experimental groups for each scaffold material were no biological agents (control), composites loaded with BM MSC plus PRP, PA, and NSE, and composites loaded with CB MSC, PRP, PA, and NSE (n = 6 per group). Each implant scaffold had the dimensions of 10 mm in diameter and 2 mm in thickness, prepared of 80% porous PCL or crosslinked collagen sponges. Scaffolds contained pores in the range of 200–500 μm. All scaffolds were sterilized and pre-coated in 10% rat PRP overnight at 4 °C. Scaffolds were then washed twice in PBS and stored at room temperature until loading. At the time of loading, solutions were prepared in two parts as described previously. *Solution A* contained cells (200,000 rat CB or BM MSC), drug-loaded NSE's (500 ng BMP2), rat PRP (100%), and PA's (10 mg/mL) and *Solution B* contained thrombin (100 units per mL) and 100 mM CaCl_2_. Solutions were pre-warmed to 37 °C and mixed just prior to loading. Each scaffold received 75 L of each formulation of Solutions A and B for a total loading volume of 150 L. After loading, each scaffold received two 5 minute treatments in a vacuum chamber over 30 minutes of attachment, and then was incubated with 1 mL osteogenic media in a 24 well plate overnight at 37 °C. To determine the number of cells not loaded into each implant, the scaffolds were moved to clean 24 well plates on the morning of surgery and the old wells were washed with PBS then supplemented 1 mL Millipore water and freeze-thawed for cell lysis. Plates were freeze thawed and analyzed by PicoGreen DNA quantification assay.

#### 2.9.2. Surgical Procedure and Implant Retrieval

Each rat received 6 implants subcutaneously; 3 on the left flank and 3 on the right. A total of of 6 male Lewis rats were used in this study, which was approved by the Institutional Animal Care and Use Committee (IACUC). Rats were anesthetized with isofluorane in O_2_, their backs shaved with an electric hair trimmer, injected with 100 μL bupivicaine (0.25% in sterile saline), and cleaned with an antiseptic lotion. A 1.5 cm incision was made vertically down the center of each rat's back and a subcutaneous pocket was made on both sides of the opening with hemostat forceps between the skin and muscle. Implant scaffolds were washed in sterile saline then inserted into their appropriate locations and then the wound was closed with 3–4 skin clips. After surgery, animals were postoperatively cared for in an oxygen-rich chamber until fully conscious then returned to their single-housing cages. After 28 days, rats were anesthetized with isofluorane in O_2_ and euthanized with increasing CO_2_. Each scaffold was removed, trimmed of connective tissue, washed in PBS, and fixed for 1 hour in 10% formalin. Scaffolds were dehydrated in a gradient of ethanol and embedded in paraffin for histological sectioning. 5 mm thick sections were stained with hematoxylin and eosin (H and E).

## 3. Results and Discussion

### 3.1. Mesenchymal Stem Cells for Bone Regeneration

**Figure 3:**
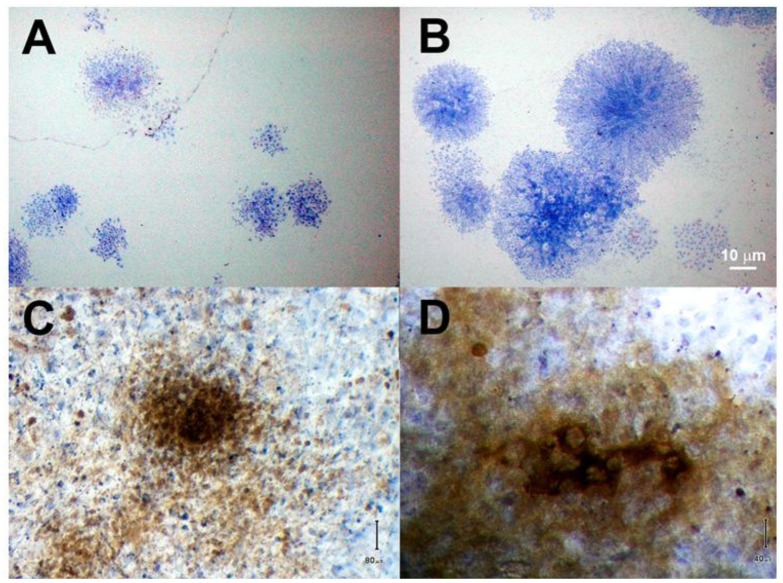
Colony forming units-fibroblast (CFU-F) from mononuclear fractions of rat bone marrow (**A**), and cortical bone (**B**). There was a significant quantitative and qualitative difference between the populations, with CB MSC occurring at higher frequency and producing larger primary colonies. Alkaline phosphatase activity (blue) and mineral deposition (brown) were stained in BM MSC (**C**) and CB MSC (**D**) after 14 days in osteogenic conditions. Both populations were ALP positive, while CB-derived cells produced far more mineral.

MSC derived from CB and BM skeletal compartments were prospectively isolated from male rats and placed into primary culture to assess CFU-F frequency. CFU-F appeared at nearly ten times the rate in purified CB MSC as in mononuclear BM MSC populations. As the fraction of mononuclear stromal cells was significantly lower in whole BM than CB, this difference in CFU-F incidence between the bulk (unpurified) populations was increased an additional 52%. The more significant finding was the difference between colony size in the populations (Figure 3). Colonies formed from CB MSC are larger in size and cell number than BM MSC at 7 and 14 days *in vitro*. The conclusion drawn from this was that not only are MSC responsible for colony formation more prevalent in the CB compartment, but the quality of cell and number of proliferated daughter cells was also greater with this cell source compared to bulk or mononucleated marrow.

The purified BM and CB MSC populations were grown in standard and osteogenic media for 14 days. ALP and Von Kossa stains for calcium are shown in Figure 3C and 3D. ALP, a marker for cell differentiation, were expressed in blue by most cells in both cultures. Mineral deposits (stained brown), however, appeared larger and denser in CB MSC cultures than BM MSC. Even with rigorous methods of prospective MSC isolation, most populations will be contaminated with other progenitor and accessory cell types from the harvest tissue. It is possible, if not likely, that the CB MSC population contained osteoblasts and other bone progenitor cell types while BM MSC included some from the hematopoetic lineage. While BM MSC are certainly capable of osteogenic differentiation, the MSC and bone progenitor cells in the CB tissue are likely more suitable for use in the formation of new bone. These findings suggest that cortical and trabecular bone tissues should be considered as a potential source of highly potent MSC for bone regeneration.

### 3.2. BMP2-Releasing Nanoporous Silicon Enclosures

The PLGA-coated, BMP2-loaded NSE were found to be uniform in size and spherical shape. They were physically sieved to obtain particles between 15–25 m in diameter. The release study revealed a nearly linear release profile, with 20%, 50%, and 90% of payload delivered *in vitro* at day 7, 24, 40, respectively. This pattern of release is advantageous compared to traditional PLGA microspheres, which exhibit a burst release profile. A continuous release of bioactive BMP2 is desirous for uninterrupted differentiation of stem cells within the compostie implant for maximum bone formation. This system provides a continuous equivalent daily dosage throughout the two week *in vitro* and four week *in vivo* studies.

### 3.3. Pre-Mineralization of Scaffolds in Vitro

Through SEM, S(P) PA nanofibers were visible on both the PA-incorporated and PA-coated scaffolds. The S(P) nanofibers were able to nucleate calcium phosphate mineral at physiological ratios on the scaffold surface, as verified by EDX, while constructs without S(P) PA did not mineralize. This mineral was observed by SEM in Figure 2B. This technology may be applied to the pre-treatment of many scaffolds prior to orthopedic implantation to create a more bioactive and biomimetic surface to promote cell attachment, differentiation, and osseointegration.

### 3.4. Scaffold Coatings and Injectable Carrier Gel for Cell and Microparticle Retention

**Figure 4:**
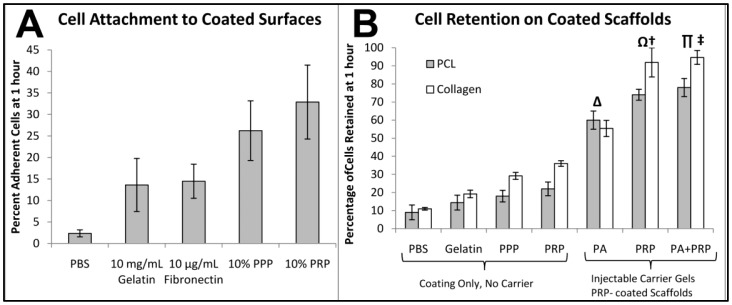
Number of CB MSC attached to bacterial grade (non-adherent) plastic coated with various protein-containing solutions after 1 hour. 10% PRP resulted in the greatest cell retention, while both PRP and PPP were superior to gelatin or fibronectin coatings (**A**). Percentage of cells retained by coated or gelled PCL (gray) and collagen (white) scaffolds after 24 h compared to MSC seeded onto tissue culture plastic. PRP proved to be the most potent coating agent, while PRP and PRP-PA gels retained greater than 90% of loaded cells in PRP-coated collagen scaffolds (**B**). PA gels were significantly greater for cell retention than any of the coatings (δ, p = 3.0 × 10^−7^). PA gels retained significantly less cells in either scaffold material than the PRP only gel (Ω, p = 0.012) or the PRP + PA gel carrier (Π, p < 0.002). Collagen retained significantly more cells than PCL scaffolds when delivered via PRP only gel (†, p = 0.023) or PRP + PA gel (‡, p = 0.010).

The studies to assess the loading efficiency of MSC onto porous scaffolds are critical for achieving maximal cell retention when placed *in vivo* for the purpose of more rapid cell proliferation within the scaffold or wound site and subsequent endogenous cell recruitment and bone formation. These studies were performed in order to optimize the **loading**, **adhesion**, and **retention** of cells within the pore network. The initial experiment tested cell adhesion onto non-adherent bacteria plates coated with PBS (control), gelatin (porcine), fibronectin (recombinant human), human PPP, or PRP (Figure 4A).

While all protein-based coating solutions displayed significantly greater cell retention than PBS, 10% PPP and 10% PRP exhibited substantially more cell adhesion to the bacterial plates. This finding indicates that PRP may be a superior, autologous (for patient specific tissue engineering applications), and potentially safer alternative coating agent than xenogenic or recombinant protein solutions. While these results were encouraging, they raised questions about the required concentration of PRP for optimal attachment and the cross-species efficacy of MSC and PRP interactions. We then examined the attachment of rat CB MSC to rat and human PRP-coated bacteria plastic at concentrations of 0.1, 1, and 10% in PBS (Figure S1 in supplemental material). It was observed that rat PRP followed the trend of human PRP at all concentrations and exhibited significantly increased attachment of cells compared to PBS controls. Further, significant differences were observed by the increase from 0.1% to 10% for both species' PRP. A subsequent experiment using rat PRP at 25% showed little increase in cell attachment over 10%. These findings are critical to the validation of rat PRP as a coating material for scaffold implants to maximize the number of loaded cells retained within the implant for *in vivo* applications.

MSC were loaded into porous PCL and collagen scaffolds as part of the optimization process to determine the best method for retaining a maximal amount of exogenous cells within a tissue engineering construct. The attachment of cells to 3D scaffolds was performed in two manners. First, MSC were seeded in PBS on to scaffolds coated with 1% gelatin, 10% PPP, or 10% PRP and allowed to adhere to the scaffold. For increased cell retention, MSC were delivered via PA, PRP, or PRP-PA gels into the pore network of scaffolds. The initial experiment summarized below in Figure 4B describes the superior effect of PRP as a pre-treatment agent to permit the greatest cell adhesion compared to PBS (negative control), gelatin, or PPP. This was in complete accord with previous 2D MSC attachment experiments.

Compared to controls (cells grown on tissue culture plastic), over 78% (PCL) and 94% (collagen) of loaded cells were retained by the PRP-PA gel carrier. The elevated cell counts from PRP and PRP-PA carriers were likely due to a combination of effects. First, fibrinogen-clotted gels were quite sturdy and did not leak liquids when perturbed with a pipette tip or spatula. Secondly, the abundance of fibrinogen and fibronectin in the plasma lends itself to higher initial cell attachments. Thirdly, the previously demonstrated proliferative or mitogenic effects of PRP on MSC may result in more cell division events during the culture period compared to controls cultured in standard media. The results of this study indicate that a synergistic effect of gelation from PRP and the peptide amphiphiles is best for retaining cells within a porous network.

**Figure 5:**
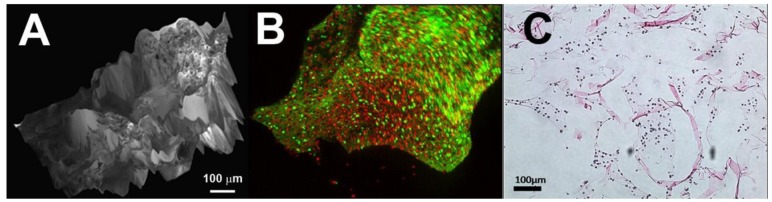
Confocal microscopy images of a collagen bioactive sponge (**A**), loaded with MSC (green) and NSE (red) (**B**). An even distribution of cells and particles was observed throughout the entirety of the scaffold. After overnight culture, histologically sectioned and stained scaffolds show cells attached to and growing throughout the scaffold (**C**).

**Figure 6:**
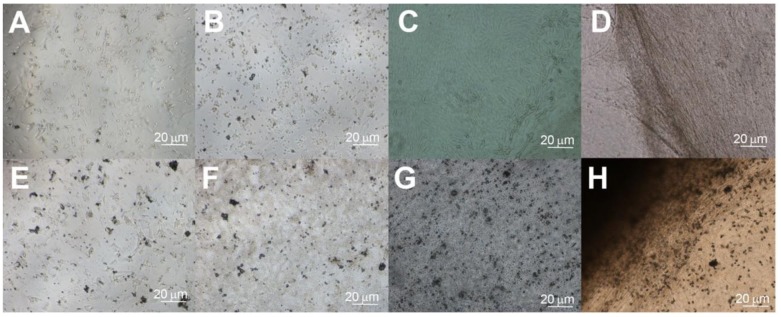
Out of phase light microscopy images of CB MSC in PRP gels in standard media at day 7 (**A**), 14 (**B**), and 21 (**C**), and osteogenic media at day 7 (**E**), 14 (**F**), and 21 (**G**). Increased cell density was observed in 2D and 3D throughout the gels with time, while accumulation of mineral deposits (black) was seen from gels cultured under osteogenic differentiation conditions. 3D PRP gels cultured for 21 days in standard (**D**) and osteogenic (**H**) media produced an abundance of cells, nodules of mineral are present throughout the 3D gel in osteogenic media. PRP gels may be considered as an osteoconductive injectable material for cell and particle delivery.

To confirm the uniform and complete distribution of cells and drug delivery particles throughout the pore network of scaffolds, fluorescently labeled MSC and NSE were injected into collagen and PCL scaffolds via the PRP-PA carrier gel. Confocal images of the 3D scaffolds and an H and E histological section of a representative composite collagen sponge are shown in Figure 5. For both material types, there was a consistent delivery of cells and particles through the entirety of the scaffold. After overnight culture at 37°C (Figure 6C), cells appeared to be attached to the surface of the scaffold itself rather than suspended or trapped within the gel. This method of injecting agents into a porous scaffold or wound repair site with an angiogenic and mitogenic (due to PRP) gelling matrix may prove beneficial to many tissue engineering functions.

### 3.5. In Vitro Mineralization—Injectable Gel Carrier

It has been previously reported that PRP may in some way impair the differentiation of MSC or retard the bone formation process *in vivo*, while the majority of studies have drawn no distinct conclusion [57,91,92,93]. To examine if the MSC were still capable of differentiation toward bone after injection with the PRP-based gel carrier, 25,000 CB MSC were loaded into alliquots of 500 L human PRP, gelled, and cultured for 21 days in standard or osteogenic media under hypoxic conditions. Differences in mineral deposition can be observed as early as Day 7, with extensive nodules of mineral by Day 21 (Figure 6). As both experimental groups were in the presence of PRP, there was considerable but equivalent cell proliferation over three weeks (303,000 cells/gel in standard media, 292,000 cells/gel in osteogenic conditions). Another advantage of PRP gels is the increased surface area upon which cells have to migrate, divide, and differentiate. Figure 6D and 6H shows sheets of MSC growing in the gels at contrasting planes of depth, with significant calcium deposits visible throughout the gel.

The amount of mineralization created by the cells was quantified by calcium assay. (Normalized calcium amounts (total calcium less the calcium present in acellular control gels) and calcium per cell values are reported in Figure S2 in the supplemental material). Significantly more mineral was produced in the presence of osteogenic media, with calcium per cell values of 81 ± 41 pg (PRP gel in standard media) *versus* 561 ± 136 pg (PRP gel in osteogenic media). These findings conclude that, at least in an *in vitro* environment, PRP does not prevent the differentiation of MSC or the desposition of a calcium phosphate throughout the extracellular matrix network and porous scaffold. Future studies will investigate the use PRP gels as an injectable osteoconductive biomaterial for MSC and/or drug-loaded particle delivery into scaffolds or directly to fracture sites. Nevertheless, these results refute the claims by some clinicians that PRP has an undesirable or inhibitory effect on bone formation in terms of its interactions with native or implanted MSC.

### 3.6. In Vitro Mineralization—Assembled Scaffolds

**Figure 7:**
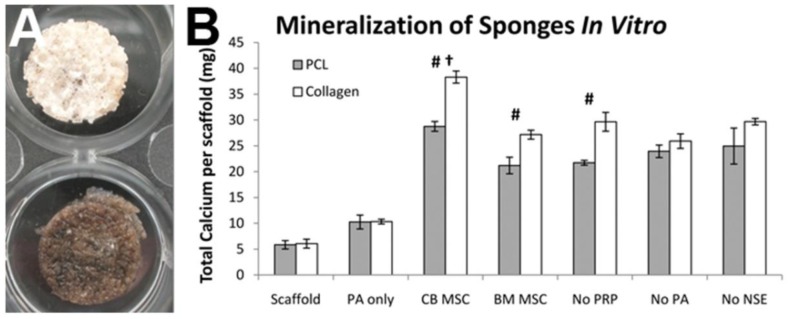
(**A**) Von Kossa stained PCL bioactive sponges after 15 in osteogenic conditions; scaffold only (top) and scaffold loaded with CB MSC, PRP, PA, and NSE (“CB MSC”, bottom); (**B**) Total calcium content per scaffold for PCL (gray) and collagen (white) scaffolds cultured for 15 days in osteogenic media (Scaffold), with S(P) and E_3_ PA (PA only), total composite formulations of cells, PRP, PA, and NSE (CB MSC or BM MSC), or total composite formulation with CB MSC without PRP (No PRP), PA (No PA), or NSE/BMP2 (No NSE). Mineralization was greater for collagen scaffolds under all conditions, with statistically significant differences (**#)** in the CB MSC (p = 3.924 × 10^−4^), BM MSC (p = 0.005), and No PRP groups (p=0.002). Significant differences in mineral production (**†**) were observed between CB and BM MSC groups on PCL (p = 0.014) and collagen scaffolds (p = 1.069 × 10^−7^).

Composite scaffolds of collagen and PCL were coated with 10% PRP then loaded with PA only, or combinations of CB/BM MSC, PRP, PA, and BMP2-releasing NSE via the fibrinogen-thrombin gel carrier. The scaffolds were cultured for 15 days in osteogenic media then assayed for number of cells and extent of mineralization. The cell counts demonstrated greater proliferation of CB MSC compared to BM MSC (15% more on PCL, 12% more on collagen scaffolds) for total composites (cells, PRP, PA, and NSE), but more importantly that both cell types proliferated at the highest rate on scaffolds comprised of collagen rather than the synthetic hydrophobic polymer. Von Kossa stains of composite scaffolds immediately indicated a considerable difference between the amounts of mineral present between experimental groups (Figure 7A). PCL and collagen sponges featuring the complete osteogenic cocktail of components stained positively for calcium ions on the surface and throughout the pores, while the acellular composites displayed small areas of darkly stained matrix sprinkled throughout the scaffold.

Mineral quantification also revealed significantly greater amounts of calcium in collagen scaffolds compared to equivalently loaded PCL (Figure 7B). The incorporation of PA (“PA only”) without cells demonstrated mineralization of the scaffolds, and their exclusion from the total composite sponges (“No PA”) resulted in a substantial drop in calcium content compared to the “CB MSC” group. Removal of PRP from the sponges only marginally decreases calcium levels, likely due to less total cells in the composite as PRP accelerates cell division prior to and during differentiation. For the groups that included MSC in the sponge, the calcium per cell values are provided in Table 3.

**Table 3:** Average calcium per cell (pg/cell) values for Composite Bioactive Sponges cultured in osteogenic media for 15 days.

Experimental Group	**PCL**	**Collagen**
**CB MSC (CB, PRP, PA, NSE)**	140.5	138.9
**BM MSC (BM, PRP, PA, NSE)**	119.6	110.5
**No PRP (CB, PA, NSE)**	132.2	148.1
**No PA (CB, PRP, NSE)**	113.4	94.9
**No NSE (CB, PRP, PA)**	133.1	141.5

For both scaffold materials, the “CB MSC”, “No PRP”, and “No NSE” groups exhibited comparable calcium per cell values. The “BM MSC” groups displayed significantly less mineralization, indicative of the superior potential of CB MSC compared to BM for bone forming and regeneration applications. As the inclusion of PA to acellular scaffold in osteogenic media caused elevated calcium content (Figure 7 and Table 3), the elimination of PA from the total composite in the “No PA” group significantly decreases the mineral present after 3 weeks. While the removal of NSE from the composite sponge did not affect the amount of calcium generated per cell, it was hypothesized to be due to the nature of the *in vitro* assay, where BMP2 has a marginalized function in the presence of a potent osteogenic media. More importantly, we take away that the inclusion of NSE (and its degradation products) cause no impairment of mineralization or MSC differentiation.

### 3.7. In Vivo Bone Formation and Angiogenesis

#### 3.7.1. Implant Preparation

**Figure 8:**
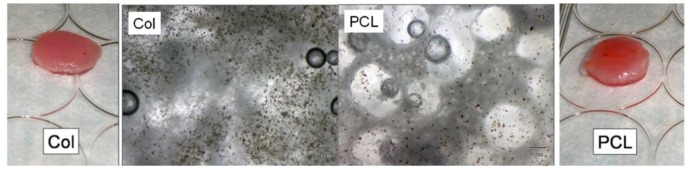
Photographs and light microscopy images of NSE/PA/PRP-loaded porous collagen (left) and PCL (right) scaffolds. NSE's appeared dark and brown under light microscope. Distribution of NSE and cells suspended in the PRP gel was observed throughout planes of depths in the interconnected pore network.

Cells, BMP2-loaded NSE, PA, PRP, and gelation agents were prepared in two solutions such that the liquids would gel when concurrently injected at 37 °C and mixed into the scaffolds' porous space. Figure 8 shows the fully loaded bioactive sponges of collagen and PCL light microscope images of the MSC and NSE within the pore network. Cells and NSE are seen to be evenly distributed throughout the porous network of each scaffold, regardless of material type.

Assuming only minimal proliferation during the overnight incubation (approximately 8 h), the average number of cells loaded in the scaffolds was 179,000 ± 6,000. This represents a loading efficiency of at least 89%, comparable to that observed during *in vitro* 3D scaffold loading experiments with PRP-PA gel carriers.

#### 3.7.2. *In Vivo* Bone Formation and Angiogenesis

**Figure 9:**
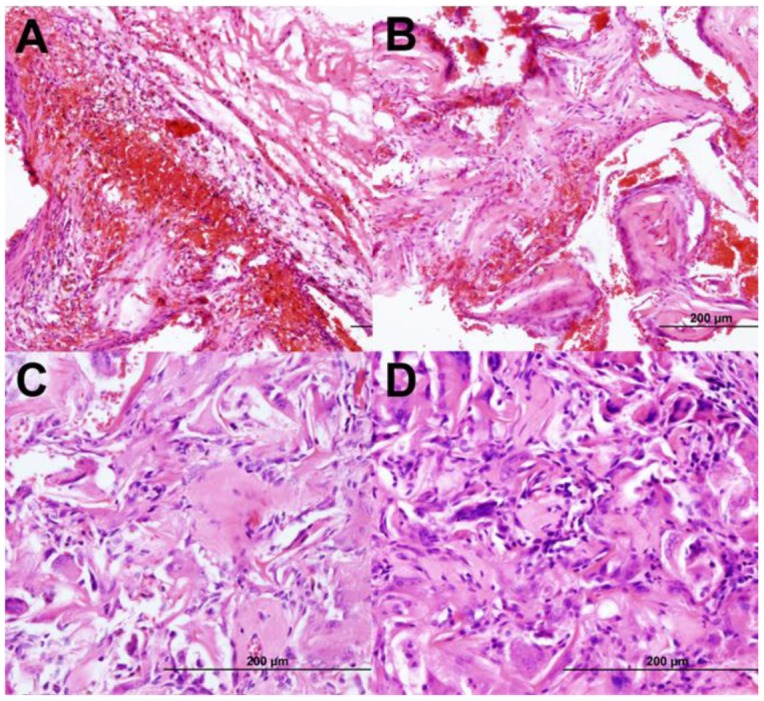
Representative H and E histological stains of PCL sponges with BM MSC (**A**) or CB MSC (**B**) and collagen sponges with BM MSC (**C**) or CB MSC (**D**) after 4 weeks *in vivo*. The empty pores are observed to be more filled with osteoid (smooth pink regions) for bioactive sponges receiving CB MSC than BM, while collagen sponges promote more bone tissue formation than PCL scaffolds.

As a pilot study for the bone-forming capacity of this composite material, loaded sponges were implanted subcutaneously on the rear flanks of male Lewis rats for 4 weeks. Previously, this method and model have been established to screen the osteogenic/osteoinductive/osteoconductive, biodegradation, and inflammatory properties of potential scaffolds and materials prior to orthotopic defect implantation [94,95,96,97]. Preliminary histological evaluation was conducted on the total composite bioactive sponges containing CB and BM MSC, PRP, PA, and NSE. H and E stains of representative sections are shown in Figure 9 below. While empty (unloaded) PCL and collagen scaffolds became filled with fibrous connective tissue, the composite bioactive sponges generated new bone of varying degrees based upon the scaffold material and stem cell type. The PCL scaffolds appeared to undergo minimal degradation in size or mass after 4 weeks, while collagen sponges did resorb at an inversely proportional rate to bone formation (*i.e.*, more osteoid correlated to less implant degradation). There were no signs of inflammatory response in or adjacent to the scaffolds.

**Figure 10:**
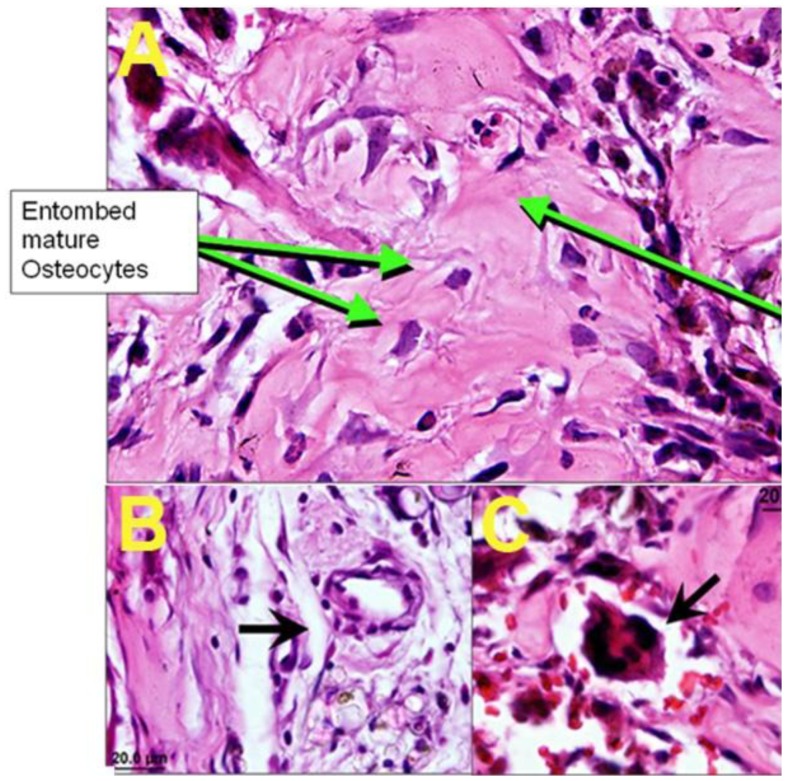
H and E histological stain of CB MSC-loaded collagen sponges after 4 weeks *in vivo* featuring entombed osteocytes within newly formed bone matrix while invading osteoclast (bone resorbing cell) was recruited from the host to remodel the tissue (**A**). The collagen sponges loaded with CB MSC, PRP, PA, and NSE generated the most blood vessels throughout the implants (**B**) and resulted in greater frequency of osteoclasts working to resorb the ectopic osteoid matrix (**C**).

Collagen-based sponges became dense neo-bone tissue and well vascularized, while PCL scaffolds possessed less total tissue within their pores. As such, the occurrence of new osteoid (bone matrix) was proportionally less in PCL sponges than collagen. The other major variable, stem cell source, proved to be equally impactful. CB-derived MSC produced significantly more osteoid throughout the implant than analogous BM MSC implants. This further emphasizes *in vitro* findings that CB MSC may be a superior and more potent source of MSC for orthopedic regenerative medicine applications. To demonstrate the quality of newly formed bone by CB MSC in the collagen-based bioactive sponges, Figure 10A exhibits maturing osteoid matrix adjacent to multi-nucleated osteoclasts (bone-resorbing cells). The presence of recruited endogenous osteoclasts was indicative that the fully loaded composite sponges were successfully osteogenic, as they would not normally be found in a subcutaneous (ectopic) site such as the rear flank muscle pouch [95,98,99,100]. In an orthotopic or fracture site, the recruitment of osteoclasts is necessary for eventual bone remodeling and strengthening [101,102,103]. Tissue engineering of bone requires a delicate balance of interplay between MSC/osteoblasts and osteoclasts in order to form structurally accurate and strong bone tissue.

In addition to new bone formed by the osteogenic sponges, blood vessels were obvious to the eye upon implant retrieval. It has been previously reported that osteopontin, present in newly forming bone, promotes angiogenesis for the transport of nutrients and waste to and from the tissue [98]. Here we find a similar trend, with an increase in vessel frequency and size correlating with the area of osteoid present in the explants. Collagen sponges appeared more thoroughly infiltrated by vasculature than PCL, which was confirmed by histological evaluation (Figure 10B). Although blood vessels and osteoclasts were present in all sponges containing MSC, PA, and NSE, the composite featuring CB MSC, PRP, PA, and NSE within a collagen scaffold produced significantly more osteoid, osteoclasts, and vessels by volume.

## 4. Conclusions

The multi-functional composite bioactive sponge generated mineralized matrix *in vitro* and induces the formation of bone and angiogenesis *in vivo*. Cell loading and attachment studies have indicated that with a PRP pre-treatment of scaffolds it was possible to retain a significant amount of seeded cells, even on a hydrophobic synthetic polymer such as PCL. Greater than 90% loading was achieved for MSC when delivered via PRP-based injectable gel, an acceptable rate for *in vivo* tissue engineering and translational applications. PA encouraged the mineralization of scaffolds *in vitro* and enhanced the retention of MSC when combined with the PRP gel. The *in vitro* differentiation study indicated that collagen was a superior substrate for cell attachment and proliferation to PCL, producing greater cell counts and more mineral under various identical conditions. The increased mineralization corresponded to higher cell numbers on the collagen sponges, as calcium content per cell values were roughly equivalent for each experimental variable. After 4 weeks *in vivo*, collagen sponges produced more osteoid, tissue integration, and blood vessel formation than analogous PCL composites. These findings, with consideration that it is the organic component of the native bone niche, conclude that collagen was a logical choice for a scaffold material to meet the biological requirements of bone regeneration. In terms of colony formation frequency and size, differentiation capacity, ability to mineralize 3D tissue engineering scaffolds, and generate new bone *in vivo*, MSC derived from cortical bone outperformed that of bone marrow. For applications that consider the use of allogenic stem cells for regenerative therapy, CB MSC should be considered as a potent cell source and potentially better alternative to the current standard, bone marrow. A novel cell and microparticle delivery system with PRP and PA was presented that retains >90% of its cargo, sets within minutes, and promotes cell proliferation and angiogenesis. Furthermore, we proved that PRP does not prevent or inhibit the osteogenic differentiation of MSC. We conclude that multi-composite bioactive osteogenic sponges comprised of a collagen matrix, CB MSC, PRP, and PA with BMP2 delivered via NSE are capable of rapidly forming osteoid and should be tested in an orthotopic fracture setting to fully evaluate its effectiveness for complete healing of non-union defects. This translation of tissue engineering concepts into clinical practice offers enormous input into the field of bone regeneration and holds potentials for translation and future change in skeletal orthopedic practice.

## Figures and Tables

**Figure S1:**
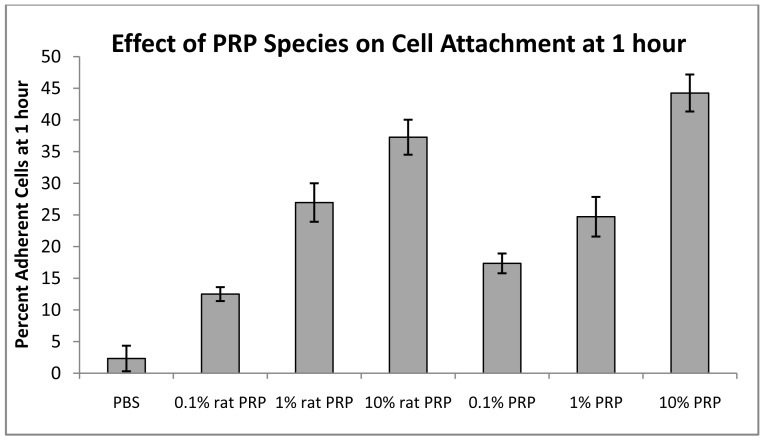
Rat CB MSC were attached to non-adherent plastic coated in PBS, rat PRP, or human PRP at increasing concentrations over 1 hour of culture. Rat PRP was effective in cell adhesion and comparable to human PRP at equivalent concentrations.

**Figure S2:**
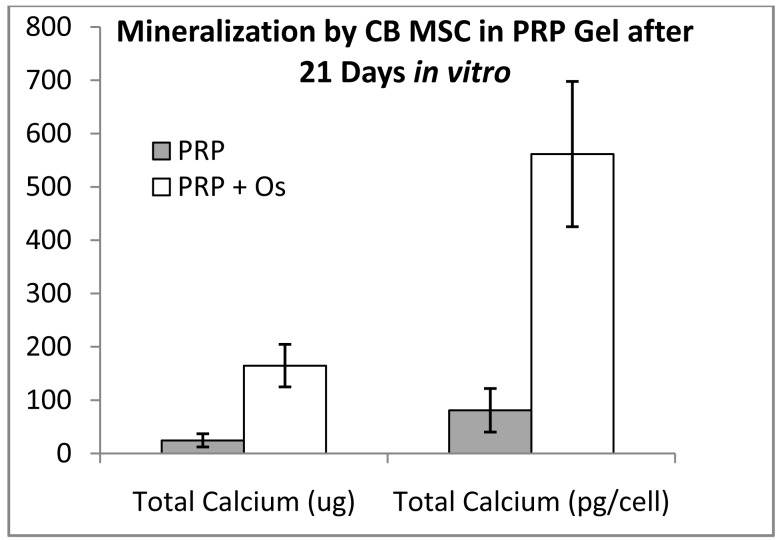
Total calcium (μg) and calcium per cell (pg/cell) generated by MSC gelled within the PRP injectable carrier after 21 days *in vitro* in standard (gray) or osteogenic (Os, white) media. PRP did not prevent the differentiation of MSC towards osteoblasts and mineral deposition.
